# Dietary resources shape the adaptive changes of cyanide detoxification function in giant panda (*Ailuropoda melanoleuca*)

**DOI:** 10.1038/srep34700

**Published:** 2016-10-05

**Authors:** He Huang, Shangmian Yie, Yuliang Liu, Chengdong Wang, Zhigang Cai, Wenping Zhang, Jingchao Lan, Xiangming Huang, Li Luo, Kailai Cai, Rong Hou, Zhihe Zhang

**Affiliations:** 1 Chengdu Research Base of Giant Panda Breeding, Sichuan Key Laboratory of Conservation Biology for Endangered Wildlife, Chengdu, 610081, China

## Abstract

The functional adaptive changes in cyanide detoxification in giant panda appear to be response to dietary transition from typical carnivore to herbivorous bear. We tested the absorption of cyanide contained in bamboo/bamboo shoots with a feeding trial in 20 adult giant pandas. We determined total cyanide content in bamboo shoots and giant panda’s feces, levels of urinary thiocyanate and tissue rhodanese activity using color reactions with a spectrophotometer. Rhodanese expression in liver and kidney at transcription and translation levels were measured using real-time RT-PCR and immunohistochemistry, respectively. We compared differences of rhodanese activity and gene expressions among giant panda, rabbit (herbivore) and cat (carnivore), and between newborn and adult giant pandas. Bamboo shoots contained 3.2 mg/kg of cyanide and giant pandas absorbed more than 65% of cyanide. However, approximately 80% of absorbed cyanide was metabolized to less toxic thiocyanate that was discharged in urine. Rhodanese expression and activity in liver and kidney of giant panda were significantly higher than in cat, but lower than in rabbit (all *P* < 0.05). Levels in adult pandas were higher than that in newborn cub. Phylogenetic analysis of both nucleotide and amino acid sequences of the rhodanese gene supported a closer relationship of giant panda with carnivores than with herbivores.

Cyanide occurs naturally as cyanogenic glycosides in a number of plants such as sorghum, linseed, clovers, grasses, cassava and bamboo, and most of those plants are food resources for both human and herbivores^1^,^2^.

However, when cyanogenic glycosides are hydrolyzed during ingestion, they produce hydrogen cyanide (HCN) that is highly toxic. HCN can inhibit the enzyme cytochrome oxidase resulting in cellular hypoxia and cytotoxic anoxia that may be fatal^3^,^4^. For human and mammals, the primary targets of cyanide toxicity are the cardiovascular, respiratory and central nervous systems. The endocrine system is also a potential target for long-term toxicity, as long-term exposure to cyanide may impair thyroid function^5^,^6^,^7^.

Nevertheless, herbivores have a physiological detoxification mechanism to convert toxic substances to less toxic metabolites that are removed from body^8^. Rhodanese (thiosulfate: cyanide sulfurtransferase, EC.2.8.1.1) plays a central role in cyanide detoxification^9^,^10^,^11^,^12^,^13^. Rhodanese is a mitochondrial enzyme that detoxifies cyanide (CN^−^) by converting it to thiocyanate (SCN^−^)^14^,^15^ Approximately 65~80% of absorbed cyanide is metabolized to less toxic thiocyanate by rhodanese in herbivorous and omnivorous mammals, and excreted in urine^16^,^17^,^18^,^19^. The rest of the absorbed cyanide is detoxified by minor pathways such as reaction with cystine to produce aminothiazoline and imino thiazolidine carboxylic acids and combination with hydroxycobalamin to form cyanocobalamin^19^,^20^.

Levels of rhodanese in tissues of animals may reflect the efficacy of the tissue cyanide detoxification function^21^. Distribution of rhodanese in different tissues appears to be tissue and species specific^22^,^23^,^24^,^25^. In most animals studied so far the level of rhodanese in different tissues is correlated with levels of exposure to cyanide^12^,^13^,^21^,^26^ and rhodanese functions in cyanide metabolism in those tissues that are apparently in direct contact with ingested cyanide^27^. Since carnivores are less likely to be exposed to cyanide through food than herbivores, they have very low levels of rhodanese in their tissues when compared with the same tissues of herbivores^12^,^28^.

Giant panda (*Ailuropoda melanoleuca*), a conservation reliant endangered species, belongs to the order Carnivora. retains a typical gastrointestinal tract of carnivorous animals^29^ and its genome codes for all necessary enzymes associated with a carnivorous digestive system^30^. However, it is well known for dietary specialization since it is a herbivorous bear with 99% of its food consisting of bamboo/bamboo shoot^31^. This specialization occurred in the late Pliocene and early Pleistocene^32^, associated with the large bamboo resources that appeared in southern China at that time^33^.

Giant pandas eat more than 60 bamboo species in the five mountainous regions they inhabit^34^. Our previous experiments showed that the staple kinds of bamboo/bamboo shoots eaten by giant panda contain corresponding cyanogenic glycoside. The average adult giant panda eats as much as 23 to 38 kg of bamboo shoots a day^31^,^35^. Given this large diet of bamboo/bamboo shoots, even if the content of cyanogenic glycosides in the bamboo/bamboo shoots is relatively low and large amounts of indigestible plant material rapidly pass through their short, straight digestive tract^36^,^37^, the giant panda is still potentially exposed to the risk of chronic food poising. Therefore, giant panda should have undergone adaptive changes in cyanide detoxification during their transition from typical carnivore to herbivorous bear.

To test that hypothesis, we investigated how much cyanogenic glycoside in bamboo shoots was absorbed daily by giant panda and whether the absorbed toxic material was converted by rhodanese into less toxic metabolites. Secondly, we compared the rhodanese expression at both transcription and translation levels and rhodanese activity in giant panda with cat and rabbit, which represented carnivores and herbivores, respectively, and between newborn and adult giant pandas. Finally, we analyzed the phylogenetic relationship of the rhodanese gene on both the nucleotide and amino acid sequence levels to test whether phenotypic changes were imprinted on a genetic basis.

## Results

### The cyanide absorption and conversion in giant pandas on the feeding trial

During the course of the 28-day feeding trial, total cyanide content in bamboo shoots was 3.2 ± 0.6 mg/kg while the corresponding content of total cyanide in feces was 1.28 ± 0.15 mg/kg for male giant pandas and 1.27 ± 0.12 mg/kg for female giant pandas, respectively (Table 1). Thus, the daily cyanide absorption was 0.52 ± 0.08 mg/ kg body mass for male giant pandas and 0.56 ± 0.05 mg/ kg body mass for female giant pandas, respectively. The daily absorption rates of cyanide in female and male giant pandas were 65.5 ± 0.9% and 64.8 ± 0.5%, respectively. The differences between female and male giant pandas were not statistically significant (Independent-Sample *T* Test, *P* = 0.259 for comparison of daily cyanide absorption /kg body mass and *P* = 0.078 for comparison of daily absorption rates of cyanide). Approximately 80% of the absorbed cyanide was metabolized to less toxic thiocyanate in the giant panda (Table 2). The amount of thiocyanate discharged in urine was 1.82 mmol/day.

### The expressions of rhodanese mRNA and protein expressions and rhodanese activity in liver and kidney of adult giant panda

**Figure 1:**
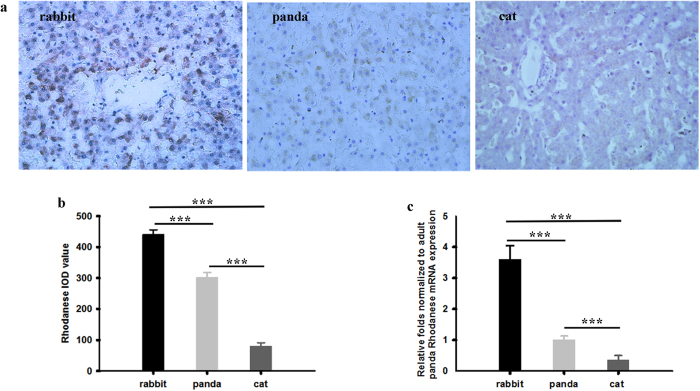
(**a**) Rhodanese protein expression in liver of rabbit, giant panda and cat. Positive staining was shown in brown. Image magnification was × 400. (**b**) Comparison of IOD sum values of the expression in liver among rabbit, giant panda and cat by One-Way ANOVA followed by Dunnet’s test. Values are mean ± SE and 441 ± 15 (n = 5), 302 ± 16 (n = 5) and 81 ± 10 (n = 5) for rabbit, giant panda and cat, respectively. (**c**) Comparison of rhodanese mRNA expression in liver among rabbit, giant panda and cat by One-Way ANOVA followed by Dunnet’s test. Values are mean ± SE and 3.60 ± 0.44 (n = 5), 1.00 ± 0.12 (n = 5) and 0.36 ± 0.14 (n = 5) for rabbit, giant panda and cat, respectively. ^***^indicates *p* < 0.001.

**Figure 2:**
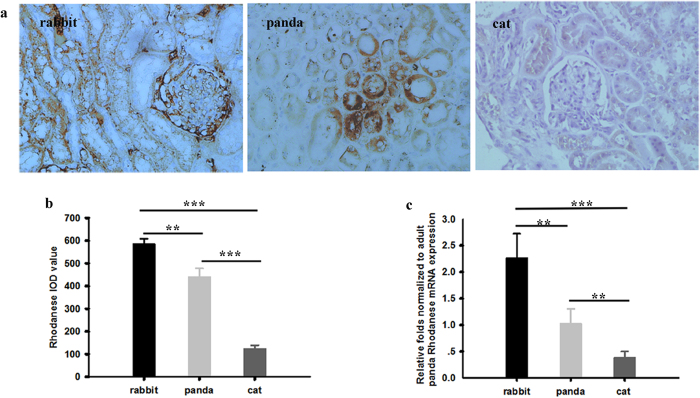
(**a**) Rhodanese expression in kidney of rabbit, giant panda and cat. Positive staining was shown in brown. Image magnification was × 400. (**b**) Comparison of IOD sum values of the expression in kidney among rabbit, giant panda and cat by One-Way ANOVA followed by Dunnet’s test. Values are mean ± SE and 586 ± 22 (n = 5), 442 ± 35 (n = 5) and 125 ± 14 (n = 5) for rabbit, giant panda and cat, respectively. (**c**) Comparison of rhodanese mRNA expression in kidney among rabbit, giant panda and cat by One-Way ANOVA followed by Dunnet’s test. Values are mean ± SE and 2.27 ± 0.46 (n = 5), 1.03 ± 0.28 (n = 5) and 0.39 ± 0.11 (n = 5) for rabbit, giant panda and cat, respectively. ** ^and^ ***indicate *p* < 0.01 and <0.001, respectively.

**Figure 3:**
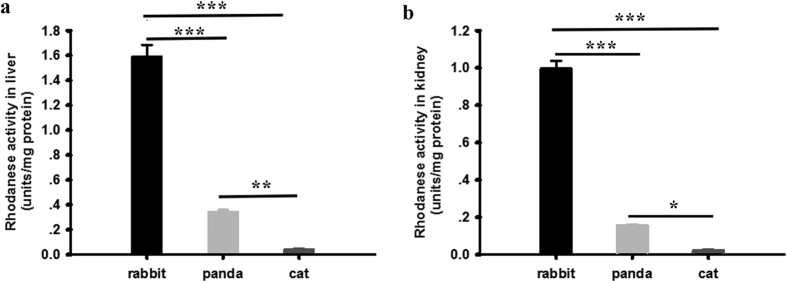
(**a**) Comparison of rhodanese activity (unit/mg protein) in liver among rabbit, giant panda and cat by One-Way ANOVA followed by Dunnet’s test. Values are mean ± SE and 0.349 ± 0.009 (n = 5), 1.595 ± 0.090 (n = 5) and 0.046 ± 0.004 (n = 5) for rabbit, giant panda and cat, respectively. (**b**) Comparison of rhodanese activity (unit/mg protein) in kidney among rabbit, giant panda and cat by One-Way ANOVA followed by Dunnet’s test. Values are mean ± SE and 0.997 ± 0.041 (n = 5), 0.156 ± 0.004 (n = 5) and 0.022 ± 0.007 (n = 5) for rabbit, giant panda and cat, respectively. *, ** ^and^ ***indicate *p* < 0.05, <0.01 and <0.001, respectively.

We measured the mRNA and protein expressions of rhodanese in liver and kidney of giant pandas by using real-Time PCR and immunohistochemistry, respectively. Rhodanese mRNA and protein expression occurred in both liver and kidney (Figs 1 and 2). Rhodanese activity in liver was 0.349 ± 0.009 unit/mg protein and in kidney was 0.156 ± 0.004 unit/mg protein (Fig. 3). These results indicated that giant panda had the rhodanese pathway for cyanide detoxification.

### Comparison of rhodanese mRNA and protein expressions and rhodanese activity among herbivore, carnivore and giant panda

To explore whether there were differences among herbivore, carnivore and giant panda, we compared the rhodanese mRNA and protein expressions and rhodanese activity. For comparison of the enzyme protein expression, we took IOD sum values of rhodanese immunostaining as indicators for evaluating the levels of rhodanese protein expression since IOD was a representative parameter to assess the immunostaining quantification, and provided a reliable and reproducible analysis of protein expression^38^. The IOD sum values of rhodanese expression in liver and kidney of giant panda were significantly lower than in rabbit (One Way ANOVA, *P* < 0.001 and <0.01 for liver and kidney, respectively) but significantly higher than in cat (One Way ANOVA, *P* < 0.001 and <0.001 for liver and kidney, respectively) (Figs 1b and 2b). Rhodanese mRNA expression in liver and kidney of giant panda were significantly lower than in rabbit (One Way ANOVA, *P* < 0.001 and <0.01 for liver and kidney, respectively) but significantly higher than in cat (One Way ANOVA, *P* < 0.001 and <0.01 for liver and kidney, respectively) (Figs 1c and 2c). Rhodanese activities in giant panda were significantly lower than in rabbit (One Way ANOVA, *P* < 0.001 and <0.001 for liver and kidney, respectively) but significantly higher than in cat (One Way ANOVA, *P* < 0.01 and <0.05 for liver and kidney, respectively) (Fig. 3a,b).

### Comparison of rhodanese mRNA and protein expressions and rhodanese activity between newborn and adult giant panda

**Figure 4:**
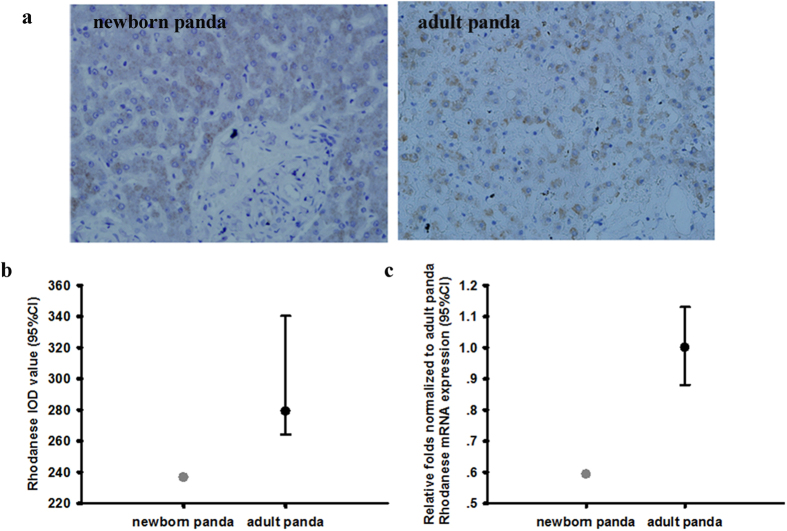
(**a**) Rhodanese protein expression in liver of newborn and adult giant panda. Positive staining was shown in brown. Image magnification was  × 400. (**b**) Comparison of IOD sum values of the expression in liver between newborn and adult giant panda. Values for newborn panda is 236 (n = 1), and for the adult giant panda is 279 ± 38 (median ± 95% CI, n = 5). (**c**) Comparison of rhodanese mRNA expression in liver between newborn and adult giant panda. Values for newborn panda is 0.593 (n = 1), and for the adult giant panda is 1.00 ± 0.13 (median ± 95%CI, n = 5).

**Figure 5:**
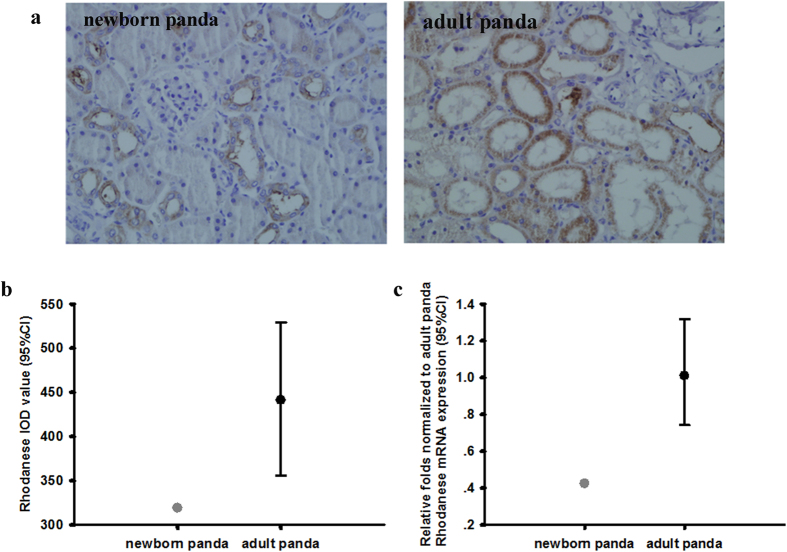
(**a**) Rhodanese protein expression in kidney of newborn and adult giant panda. Positive staining was shown in brown. Image magnification was × 400. (**b**) Comparison of IOD sum values of the expression in kidney between newborn and adult giant panda. Values for newborn panda is 319 (n = 1) and for the adult giant panda is 441 ± 86 (median ± 95% CI, n = 5). (**c**) Comparison of rhodanese mRNA expression in kidney between newborn and adult giant panda. Values for newborn panda is 0.42 (n = 1), and for the adult giant panda is 1.01 ± 0.29 (median ± 95%CI, n = 5).

**Figure 6:**
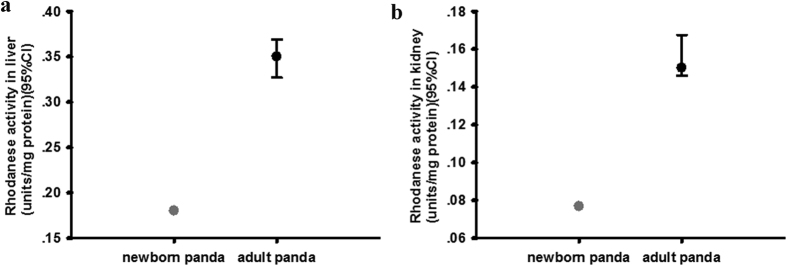
(**a**) Comparison of rhodanese activity (unit/mg protein) in liver between newborn and adult giant panda. Values for newborn panda is 0.180 (n = 1), and for the adult giant panda is 0.350 ± 0.021 (median ± 95%CI, n = 5). (**b**) Comparison of rhodanese activity (unit/mg protein) in kidney between newborn and adult giant panda. Values for newborn panda is 0.077 (n = 1), and for the adult giant panda is 0.150 ± 0.011(median ± 95%CI, n = 5).

To investigate whether there were differences in rhodanese mRNA and protein expressions and rhodanese activity in different aged giant panda, we compared newborn and adult giant pandas. Rhodanese mRNA and protein expression occurred in liver and kidney in newborn giant panda (Figs 4a and 5a). The IOD sum values of rhodanese and the mRNA expressions in liver and kidney of newborn giant panda were all lower than that in adult giant panda (for IOD sum values in liver and kidney, respectively; Figs 4b and 5b; and for the mRNA expression levels in liver and kidney, respectively; Figs 4c and 5c). The rhodanese activity in liver and kidney of newborn giant panda was also lower than that in adult giant panda (for liver and kidney, respectively; Fig. 6a,b).

### Phylogenetic analysis of rhodanese gene of different species of animals

**Figure 7:**
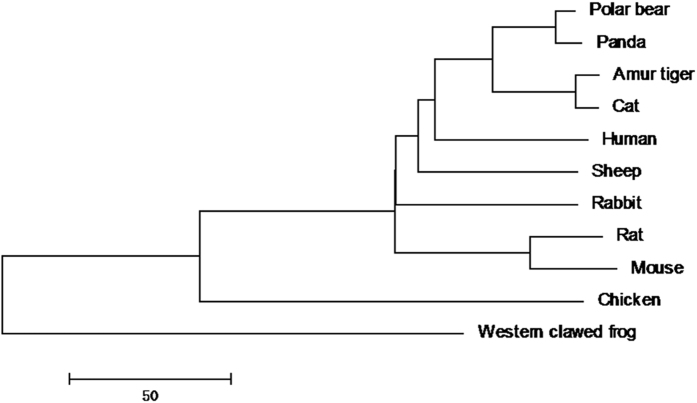
The phylogenetic NJ tree of rhodanese gene among 11 species, rooted by “Western clawed frog”. The phylogenetic tree revealed that giant panda was most closely related to the polar bear among all studied 11 species.

Based on the nucleotide sequences of rhodanese gene, the phylogenetic tree revealed that giant panda was most closely related to the polar bear among all studied species (Fig. 7). After being translated into amino acid sequences, the same phylogenetic relationship existed.

## Discussion

Large herbivores such as elk (*Cervus elaphus*)^39^ and white-tailed deer (*Odoco ileus virginia nus*)^40^ digest around 70% of dry matter while giant pandas digest only ∼17% of dry matter consumed^31^. This is because the giant panda has a typical gastrointestinal tract of carnivorous animals^29^ with much reduced segment of the small intestine and absence of caecum^41^. Thus, there are no sacculations or compartments to retain digesta for microbial fermentation in giant panda gut and the large amounts of indigestible plant material rapidly pass through their short, straight digestive tract. Thus, we found that only about 65% of cyanide in bamboo shoots was absorbed in adult giant panda in this study.

Even so, an adult giant panda absorbed an average of 54.8–66.1 mg of cyanide a day (average 0.52–0.56 mg/kg body mass) (Table 1). That amount is almost a fatal dose for a human. Thus, cyanogenic glycosides in the bamboo/bamboo shoots would have a chronic poisoning effect on giant panda if the animals had no effective detoxifying mechanism for cyanide.

Our analysis of urinary thiocyanate revealed that approximate 80% of the absorbed toxic cyanide was metabolized to less toxic thiocyanate (Table 2). The results indicate that giant pandas developed an effective anti-toxic mechanism that protected them during the transition from typical carnivore to herbivorous bear. The results also indicate that the major pathway for cyanide detoxification in giant panda was the rhodanese pathway as it was in other herbivorous and omnivorous mammals^16^,^17^,^18^,^19^.

As liver and kidney are the richest source of rhodanese and the major sites of cyanide detoxification in most animals^42^,^43^,^44^, we investigated the expression and activities of the enzyme in the two organs in this study. Enzyme expression at both transcription and translation levels in giant panda were significantly higher than that in the cat (a carnivore) but lower than in the rabbit (a herbivore) (Figs 1 and 2). The species differences in enzyme expression also occurred in the enzyme activity in which the activity in both liver and kidney of giant panda was lower than that of rabbit but higher than that of cat (Fig. 3). Enzyme activity in liver and kidney of cat determined in our study was the same as a previous report on cat^28^. Enzyme activity in liver and kidney of rabbit was also similar to that reported by Aminlari^45^.

As a carnivorous animal, the cat is less likely to be exposed to cyanide through food, and little absorbed cyanide needs to be detoxified by rhodanese, hence very low levels of rhodanese mRNA and protein expression and rhodanese activity in its tissues are expected when compared with giant panda and rabbit. In contrast, herbivores appear to have evolved mechanisms to tolerate phytotoxins^8^,^46^. As a typical herbivore, the rabbit has accommodated to feed on cyanogenic plants all the time. It turned out as expected that rabbits showed the highest rhodanese mRNA and protein expressions and rhodanese activity when compared with other species in our study.

It is also known that exposure to toxins may increase the ability of animals to metabolize the compound^8^. Our finding that the rhodanese expression and rhodanese activity in both liver and kidney in giant panda were significantly higher than that in the carnivorous cat suggests that the repeated exposure to cyanogenic bamboo/bamboo shoots in the herbivorous bear may have introduced a response to increase the rhodanese expression and activity during their evolution.

We further tested this hypothesis by comparing a newborn giant panda with adult giant pandas. Early in life, the newborn panda’s only food intake is milk from mother and little cyanide is absorbed. After about 1 year, the giant panda lives on a bamboo-dominated diet and the absorbed cyanide needs be detoxified. Thus, the rhodanese expression and activity of the adult giant pandas were increased when compared to the newborn panda (Figs 4, 5 and 6). The addition of bamboo to the diet of the young giant panda probably induces expression of the rhodanese gene.

Two millions years ago, when their prey became extinct, giant pandas suffered from a scarcity of alternative food sources^31^,^32^. Bamboo offered an abundant and readily accessible food source throughout that year, and giant pandas began to feed on bamboo, since there was no other food available to ensure their survival. As we described above, rhodanese function is correlated with levels of exposure to cyanide. The repeated exposure to cyanogenic bamboo/bamboo shoots in giant panda may have been inducing a response to increase the rhodanese expression and activity during their evolution. Over time, giant pandas were selected to increase that production until today they convert 80% of the absorbed cyanide into thiocyanate.

Giant pandas have combined morphological, behavioral, physiological and other evolutions to adapt to environmental changes a long time ago^47^,^48^. For examples, the pseudothumb, the structure of skull and mandible as well as large and flat teeth are all typical morphological adaptations to a specialized diet^48^,^49^,^50^,^51^. In addition, adaptations to the low energy diet, the giant panda has evolved a suite of behavioral and physiological adaptations including low physical activity levels and reduced sizes of some high metabolism organs to further minimize energy expenditure^52^. In our study, we found that there was a rhodanese functional adaptation in response to changes in dietary resources in giant panda although the animals maintain the genetic requirements for being purely carnivorous^30^. In the next step, the molecular mechanisms by which dietary changes to eat cyanogenic bamboo triggered the changes in rhodanese function need further studies.

Red panda (*Ailurus fulgens*) and giant pandas are mammalian carnivores by phylogeny but herbivores by diet. Both them experienced a dietary switch from carnivores to highly specialized bamboo eaters^53^. Based on our results from giant panda, we speculate that the red panda may also undergo the similar rhodanese functional adaptation as the same as giant panda. However, the speculation needs another study.

In summary, we found that 1) about 65% of cyanide in bamboo shoots was absorbed in adult giant panda; 2) approximately 80% of absorbed cyanide was metabolized to thiocyanate that was excreted in the urine; 3) rhodanese expression at both transcription and translation levels and rhodanese activity in both liver and kidney in giant panda were lower than that in rabbit but higher than that in cat; 4) the corresponding levels in both liver and kidney of newborn panda were lower than that in adult giant pandas and 5) the phenotypic adaptation of rhodanese has not been imprinted on the nucleotide sequences.

The present study, for first time, provides strong evidence that there was a rhodanese functional adaptation in response to changes in dietary resources in giant panda. The rhodanese detoxifying function changes may be an important adaptation process of many known or unknown adaptations related to morphology, behavior and physiology that allow giant pandas to survive until today.

## Materials and Methods

### Animal feeding trial and sample collection

We measured the total cyanide in samples of bamboo shoots and feces and thiocyanate levels in urine in 20 adult healthy giant pandas at the Chengdu Research Base of Giant Panda Breeding (Research Base). During the course of a 28-day feeding trial, bamboo shoots and clean drinking water were provided ad lib. Bamboo shoots used in the trial were from *Chimonobambusa szechuanensis* and gathered from the habitat of free giant pandas in Dujiangyan, Sichuan province. We recorded intake of bamboo shoots and feces and urine output for each study animal daily. Every 4 days, we sampled 500 g of bamboo shoots chosen casually from the food, about 500 g of feces, and 2 mL of urine from each animal in the morning. Following collection, we immediately measured total cyanide in bamboo shoots and feces, and stored urine samples at −20 °C until analysis.

To determine the rhodanese expression and activity in liver and kidney, we purchased 5 healthy New Zealand white rabbits (10 months old) and 5 healthy domestic cats (1–2 years old) from Animal Experiment Center, Sichuan University. We sacrificed rabbits and cats by cervical dislocation under light ether anesthesia. We quickly excised their liver and kidney and rinsed them in cold physiological saline. We separated organs into 2 portions: we immediately fixed one portion in 10% formol saline for immunohistochemical analysis, and sliced the other portion into thin sections and stored it in liquid nitrogen for real-time RT-PCR and enzyme activity assay.

We collected samples of liver and kidney of adult giant panda from 4 animals that died natural deaths at the Research Base (Stubook numbers 287, 297, 373 and 718) and 1 free giant panda rescued from wild that then died from intestinal obstruction. We also obtained samples from one 9-day-old giant panda that died from trauma at the Research Base. We treated liver and kidney samples of giant pandas as the same as those of rabbits and cats described above.

This study was approved by the Research Base. The methods used were in accordance with the approved guidelines of the institution and followed all regulations of the Research Base. Permission to conduct this research was given by the Director of the Research Base after consultation with the Research, Husbandry and Veterinary Departments. The research protocol and handling procedures were approved by the Directors and staff of the each of those departments.

### Measurement of total cyanide

We determined the cyanide level in bamboo shoots or corresponding feces samples according to the method described by Surleva^54^. We first drew a calibration graph by preparing standard solutions of CN^−^ at concentrations of 0.02, 0.04, 0.08, 0.1.0.2, 0.4 and 0.8 μg/mL by adding appropriate volumes of cyanide solutions at concentration of 20 μg CN^−^/mL (potassium cyanide dissolved in 0.01 M NaOH) to 1 mL of 2% Na_2_CO_3_. We added 0.5 mL of ninhydrin solution (5 mg/mL ninhydrin in 2% Na_2_CO_3_) to each standard cyanide solution, homoginized the mixture and incubated it for 15 min for color development. We prepared the blank in the same way as above, except that 1 mL of 2% Na_2_CO_3_ without CN^−^ instead of 1 mL 2% Na_2_CO_3_ containing CN^−^ was added. We measured UV-Visible absorption of the reaction product (Cyanide-ninhydrin adducts) of the different concentrations of cyanide using an UV/Vis Spectrophotometer (SURGISPEC SM735, Surgical Medical, England) at 485 nm.

We cut samples of bamboo shoots or feces into small sections and ground them in liquid nitrogen. We measured total cyanide in the samples by adding 0.1 g of the ground sample into a standard volumetric flask (5 mL) and making up volume to mark with 0.1% NaHCO_3_. We sonicated samples for 20 min in a water bath and centrifuged the mixture at 10,000 rpm for 10 min. We pipetted the supernatant with an automatic pipette, two aliquots (40 μL each), added 1 mL of 2% Na_2_CO_3_ and 0.5 mL ninhydrin solution, allowed 15 min for color development and measured absorbance at 485 nm. We expressed total cyanide content as mg HCN/kg.

### Measurement of urinary thiocyanate

We determined thiocyanate levels in giant panda urine by using an improved method described by Lundquist^55^. Briefly, we applied aliquots of urine (500 μL) diluted with 5.0 mL of 1 M NaOH to columns (2.5 × 0.7 cm) of Amberlyst A-21. We washed the columns 3 times with 5 mL of double-distilled water before eluting thiocyanate by adding 8 mL of 1 M sodium perchlorate. We acidified aliquots of 4 mL of elutes with 0.2 mL of 0.35M acetic acid, and chlorinated for 2 min with 0.1 mL of 50 mM sodium hypochlorite. Then, we added 0.6 mL of color reagent (a mixture of isonicotinic acid and 1, 3-dimethylbarbituric acid) to the treated urinary aliquots. At the same time, we prepared a reagent blank in which urine was replaced with double-distilled water. We performed assays in duplicate. We read thiocyanate concentrations in the samples against a standard curve with known concentrations of potassium thiocyanate. The thiocyanate levels in giant panda urine were expressed as mmol SCN^−^/L.

### Real time RT-PCR

We extracted total RNA using Trizol reagent (Invitrogen, Carlsbad CA, USA). We assessed quality and quantity of the isolated RNA with a Qubit^®^ Fluorometer (Invitrogen, Carlsbad CA, USA) and 1% TBE-Agarose gel electrophoresis, followed by reverse transcription. The 20 μL reverse transcription reaction system comprised the following: 2 μg of total RNA, 1 μL of Oligo (dT) 15 Primer, 10 μL of nuclease-free water, 1 μL of M-MLV Reverse Transcriptase, 1.6 μL of nuclease-free water, 0.4 μL of Recombinant RNase Inhibitor, 4 μL of 5 × reaction buffer, 2 μL of MgCl_2_ (25 mM), and 1 μL of PCR Nucleotide Mix. We performed the reaction procedure under the following conditions: denaturation for 5 min at 70 °C, annealing for 5 min at 25 °C, extending annealing for 60 min at 42 °C, inactivated reverse transcriptase for 15 min at 70 °C, and then storage at 4 °C.

We measured mRNA expression level of rhodanese using a 7500 Real-Time PCR System with a 20 μL reaction system containing the following: 1 μL of cDNA, 10 μL of 2 × SYBR Ssofast Evagreen^®^ master mix, 1 μL of each gene-specific primer (100 nM). We performed the reaction procedure as follows: 1 cycle of 95 °C for 30 s, 39 cycles of 95 °C for 5 s, 60 °C for 1 min and 1 cycle of 95 °C for 15 s, 60 °C for 60 s, 95 °C for 30 s, and 60 °C for 15 s. GAPDH was used as a house-keeping gene. We obtained sequences of rhodanese and GAPDH genes for rabbit, giant panda and cat from NCBI database. The primers that we chose from the highly conserved regions for the three species were designed through Oligo 6.0 and Primer 5.0 as rhodanese: 5′-GCGTCGCCCTACGAGATGATG-3′ (forward) and 5′-TTGAGCAGGGAGCGGTCCAG-3′ (reverse) and GAPDH: 5′-TGTCAGCAATGCCTCCTGTA-3′ (forward) and 5′-TTTCCGTTCAGCTCAGGGAT-3′ (reverse), and synthesized by Invitrogen Corporation.

To evaluate the relative quantification of mRNA expression, we normalized the cycle threshold (C_T_) values of rhodanese to the C_T_ values of the GAPDH gene and presented results as fold changes of 2 ^−^ ^∆∆CT^. We used the adult giant panda group as control and normalized each transcript level to adult giant panda rhodanese. We calculated relative mRNA expression of the rhodanese in each group using the following equations: ∆C_T_ = C_T (*Rhodanese*) _– C_T(GAPDH),_ ∆∆CT = ∆C_T(treated group)_ – ∆C_T(control group)_.

### Immunohistochemistry

We fixed samples of liver and kidney tissues in formalin and embedded them in paraffin. We prepared 5 μm sections, de-paraffinized with Xylene for 10 min and rehydrated in descending ethanol gradient (100–96–70%) solutions. Afterwards, we blocked endogenous peroxidase in the sections for 10 min with 3% H_2_O_2_ followed by an antigen retrieval in sodium citrate buffer (pH = 6.0) at 95 °C for 30 min. After 30 min of cooling, we blocked the slides with 10% goat serum (Abcam, UK) and 1% bovine serum albumin (Jackson Immunoresearch, U.S.A.) in PBS for 1 h at room temperature. Then we incubated the sections with 5 μg/mL of a polyclonal rabbit anti-TST primary antibody (Abcam, UK, CAT# ab60128) in a PBS buffer containing 10% goat serum and 1% BSA overnight at 4 °C. After washing four times with PBS, we incubated the sections with 1: 500 of an anti-rabbit antibody conjugated with horseradish-peroxidase (Abcam, UK; CAT# ab6721) for 1 h at room temperature. After a further four washes, we visualized binding sites of the primary antibody with 3, 3′-diaminobenzidine substrate (DAB) (Vector Laboratories, U.S.A.; CAT# SK-4105). Finally we counterstained slides with haematoxyline, dehydrated them in ascending ethanol (70–96–100%) and Xylene, and mounted them with Petrex non-aqueous medium. Negative controls were achieved by the omission of the primary antibody.

We analyzed the degree of rhodanese expressions by a method of integrated optical density (IOD). We excluded scores for the highest and lowest regions for each slide and calculated the average region score from the remaining 10 regions. For comparison purposes, we normalized the IOD value to the entire measured area by calculating IOD/0.25 mm images captured by a relevant software (Olympus DP25), and calculated the IOD sum value using Image-Pro Plus 6.0 software (Media Cybernetics, USA).

### Determination of rhodanese activity

We prepared liver and kidney extracts by freezing the samples in liquid nitrogen, homogenizing them with a hand homogenizer, and suspending the homogenates in 25 mM sodium phosphate (pH 7.2). We centrifuged suspensions for 15 min at 4,000 × *g*, and used supernatants as source of the enzyme. We assayed rhodanese activity by the modified method of Sörbo^56^. The reaction mixture contained 16.8 mM sodium thiosulphate, 40 mM glycine buffer, pH 9.2, 167 mM KCN and 50 μL enzyme solution in a final volume of 4.0 mL. We carried out the reaction for 15 min at 37 °C and stopped it by adding 0.5 mL 38% formaldehyde. We added formaldehyde in control tubes before the addition of enzyme solution. We determined concentration of thiocyanate as follows: samples were mixed with 1 mL ferric nitrate solution containing 0.025 g Fe(NO_3_)_3**·**_ 9H_2_O in 0.74 mL water and 0.26 mL concentrated nitric acid. We measured absorbance at 460 nm against a blank containing all reagents, except that 50 μL water was used instead of enzyme solution. We obtained concentration of thiocyanate formed from a standard curve produced by treating solutions containing different concentrations of thiocyanate as described above. The unit of enzyme activity was micromole of thiocyanate formed per minute at pH 9.2 and 37 °C. We reported rhodanese activity as specific activity (unit/mg protein) in which total protein was assayed according to Lowry^57^ using bovine serum albumin as a standard.

### Phylogenetic analysis of rhodanese gene

We retrieved the coding sequences of rhodanese genes from GenBank for 11 species including western clawed frog [Xenopus (Silurana) tropicalis (NM_001103038)], human [Homo sapiens (NM_003312)], Norway rat [Rattus norvegicus (NM_012808)], chicken [Gallus gallus (NM_001167731)], house rat [Mus musculus (NM_009437)], polar bear [Ursus maritimus (NW_007907256.1)], amur tiger [Panthera tigris altaica (NW_006712392.1)], domestic cat [Felis catus (NC_018729.2)], giant panda [Ailuropoda melanoleuca (NW_003217342.1)], rabbit [Oryctolagus cuniculus (NC_013672.1)] and sheep [Ovis aries (NC_019460.1)]. After conducting the codon-based multiple alignments, we constructed the Neighbor-Joining (NJ) tree by using the MEGA6^58^, which was out grouped by western clawed frog.

### Data analysis

We performed statistical analysis by using the SPSS software package (SPSS Inc., Chicago, IL, USA). We expressed data of feeding trial as mean ± SD and compared female and male giant panda groups by Independent-Sample *T* Test. We expressed data of rhodanese mRNA and protein expressions and rhodanese activity as mean ± SE and analyzed the data by One-Way ANOVA followed by Dunnet’s test for comparing the results among rabbit, cat and giant panda groups. We showed the newborn panda data as just the one value and a 95% confidence interval value for the adult giant panda data for comparison. *P* < 0.05 indicates a statistically significant difference.

## Additional Information

**How to cite this article**: Huang, H. *et al*. Dietary resources shape the adaptive changes of cyanide detoxification function in giant panda (*Ailuropoda melanoleuca*). *Sci. Rep.* **6**, 34700; doi: 10.1038/srep34700 (2016).

## Figures and Tables

**Table 1:** Feeding trial for cyanide absorption by giant pandas at the Chengdu Research Base of Giant Panda Breeding.

Sex	Age (Mean ± SD)	Mass (Mean ± SD)	Daily bamboo shoot intake (Mean ± SD, kg/d)	Cyanide in bamboo shoot (Mean ± SD, mg HCN/kg)	Daily cyanide discharged in excrement (Mean ± SD, mg HCN/d)	Daily total cyanide absorption (Mean ± SD, mg HCN/d)	Daily absorption rate of cyanide (%) (Mean ± SD)	Daily cyanide absorption /kg BW (Mean ± SD, mg HCN /kg BW)
Female (*n* = 12)	12.8 ± 4.3	98.4 ± 5.6	26.1 ± 3.5	3.2 ± 0.6	28.8 ± 4.1	54.8 ± 7.3	65.5 ± 0.9	0.56 ± 0.05
Male (*n* = 8)	13.6 ± 6.0	126.3 ± 10.7	31.8 ± 7.0	3.2 ± 0.6	35.7 ± 7.5	66.1 ± 15.0	64.8 ± 0.5	0.52 ± 0.08
*P*	0.736	0.001	0.028		0.017	0.037	0.078	0.259

**Table 2:** Feeding trial for the absorbed cyanide converted to thiocyanate(SCN^−^) in giant pandas at the Chengdu Research Base of Giant Panda Breeding.

Panda numbers	Daily urine output (Mean ± SD, L)	Urinary SCN^−^ concentration (Mean ± SD, mmol/L)	Daily SCN^−^ discharged in urine (Mean ± SD, mmol)	Daily cyanide (CN^−^) absorption (Mean ± SD, mmol)	The rate of cyanide (CN^−^) converted to SCN^−^(Mean ± SD, %)
*n* = 20	3.0 ± 0.9 (1.8 ~ 5.0)	0.60 ± 0.27 (0.36 ~ 1.0)	1.82 ± 0.39 (1.06 ~ 2.92)	2.28 ± 0.47 (1.40 ~ 3.58)	79.7 ± 0.02 (75 ~ 83)
